# The correlation between types of posterior upper rotator cuff tears and intramuscular fat infiltration based on magnetic resonance imaging: A retrospective observational study

**DOI:** 10.3389/fbioe.2022.859174

**Published:** 2022-08-23

**Authors:** Xiao-Kun Yu, Jia-Xi Cao, Lei Li, Wen-Bin Guo, Le Zhang, Jin-Xing Li

**Affiliations:** Department of Radiology, The Fifth Centre Hospital of Tianjin, Tianjin, China

**Keywords:** magnetic resonance imaging, rotator cuff injury, shoulder joint, fat infiltration, tear range

## Abstract

**Objective:** This study investigated the correlation between types of posterior upper rotator cuff tears (RCTs) and intramuscular fat infiltration (FI) in magnetic resonance imaging (MRI).

**Methods:** The shoulder joints of 50 adults with a full-thickness posterior upper RCT diagnosed by MRI, from January 2019 to December 2021, were retrospectively analyzed. The patients were divided into three groups according to tear type: 1) an L-shaped tear group; 2) a crescent/U-shaped tear group; 3) a complete tear group. The correlation among age, gender, tear range, trauma history, and the duration of clinical symptoms was analyzed. The MRI images were used by two musculoskeletal imaging physicians to evaluate the type and range of tears, the Goutallier grade of the supraspinatus and infraspinatus, and the correlations and reliability were analyzed.

**Results:** Differences in the tear range (*p* < 0.001) and the Goutallier grade of the infraspinatus muscle (*p* = 0.036) among the L-shaped, crescent/U-shaped, and complete tear groups were statistically significant; however, differences in the Goutallier grade of the supraspinatus muscle was not statistically significant (*p* = 0.356). In the crescent/U-shaped tear group, age was significantly correlated with the Goutallier grade of the supraspinatus muscle (RS = 0.720, *p* = 0.029) and the infraspinatus muscle (RS = 0.713, *p* = 0.032). In the complete tear group, tear range was significantly correlated with the Goutallier grade of the supraspinatus muscle (RS = 0.801, *p* = 0.001) and the infraspinatus muscle (RS = 0.802, *p* = 0.001). The Goutallier grades of the supraspinatus muscle (kappa, 0.489) and the infraspinatus muscle (kappa, 0.424) presented with interobserver consistency.

**Conclusion:** The type of posterior upper RCT correlates with the degree of FI. There is a positive correlation between the FI of crescent/U-shaped full-thickness RCTs and age. Additionally, the range of complete tears in the posterior upper RC has a positive correlation with FI.

## 1 Introduction

Rotator cuff tears (RCTs) are common in adults, and surgical rotator cuff repairs (RCRs) have shown a yearly upward trend (Jain et al., 2014). Although a large number of RCR operations are performed annually, the incidence of asymptomatic RCTs remains high (Frank et al., 2018). Very little is known about the clinical differences associated with asymptomatic patients with an RCT. Existing studies considered the type of RCT to be an important factor for indicating the presence of typical clinical symptoms involving the shoulder joint; accordingly, corresponding surgical methods and prognoses have been formulated (Davidson and Burkhart, 2010; Keener et al., 2015; Sela et al., 2015). For RCTs that present with significant clinical symptoms, particularly those involving the anterior part of the supraspinatus tendon, particularly significant damage may be present related to shoulder function and the risk of tear progression, making early surgical intervention important (Kim et al., 2010).

The supraspinatus and infraspinatus muscles form the posterior upper RC. However, recent autopsy studies showed that the insertion footprint of the supraspinatus tendon was small and located in the anteromedial area of the greater tubercle of the humerus. The insertion footprint of the infraspinatus tendon is relatively large, spanning the middle surface and the anterolateral area of the upper surface (Mochizuki et al., 2008; Nimura et al., 2012). The RC is a fiber band that extends in the fore-and-aft directions; it is connected to the posterior upper RC in the vertical direction and crosses the anterior edge of the supraspinatus tendon and the posterior inferior area of the infraspinatus tendon (Namdari et al., 2014; Chen et al., 2020). The segments of the supraspinatus and infraspinatus tendons on the outside of the RC are adjacent to the front and back of the RC and are collectively known as the “crescent” of the RC (Podgórski et al., 2019).

Most RCTs involve the posterior upper part. The most common full-thickness RCT type is a crescent tear (approximately 40%), followed by L-shaped (approximately 30%) and U-shaped (approximately 15%) tears; incidences involving a complete tear of the supraspinatus and infraspinatus tendons are low (Melis et al., 2009; Lädermann et al., 2016). Imaging studies revealed that the RCT was characterized by uneven FI, where a wide range FI was mainly related to a full-thickness RCT but not to a partial thickness RCT (Mellado et al., 2005; Deniz et al., 2014). In addition, a larger range for a full-thickness RCT generally showed a higher FI trend compared with smaller full-thickness tears (Lee et al., 2015). However, the correlation between the type of RCT and fat infiltration (FI) in the posterior upper RC muscle is unclear. The purpose of our study was to determine whether there was a statistical difference between different tear types in the posterior upper RC and the degree of intramuscular FI. We hypothesized the presence of a positive correlation in this regard.

## 2 Methods

### 2.1 Study design and grouping

The study population of this retrospective study comprised adult men and women ≥18 years old who underwent shoulder magnetic resonance imaging (MRI) from January 2019 to December 2021 in our hospital. All cases meeting the inclusion and exclusion criteria were included in this study and data were collected by searching the picture archiving and communication system (PACS) of the medical institution.

**FIGURE 1:**
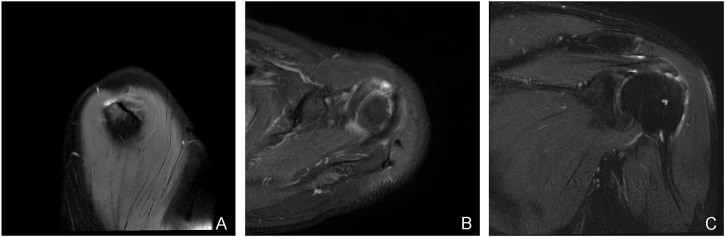
A typical case of an L-shaped tear: a 71-year-old man with diffuse tendinitis of the supraspinatus tendon in the left shoulder and a localized full-thickness tear of the tendon anterior to the tuberosity insertion. Slightly high signal intensity can be seen in the supraspinatus tendon, and an L-shaped watery tear signal can be seen in the anterior tendon at the insertion point of the humeral tubercle. **(A)** the oblique sagittal view of T2 lipid-suppressing sequence; **(B)** the axial view of proton density sequence; **(C)** the oblique coronal view of T2 lipid-suppressing sequence.

**FIGURE 2:**
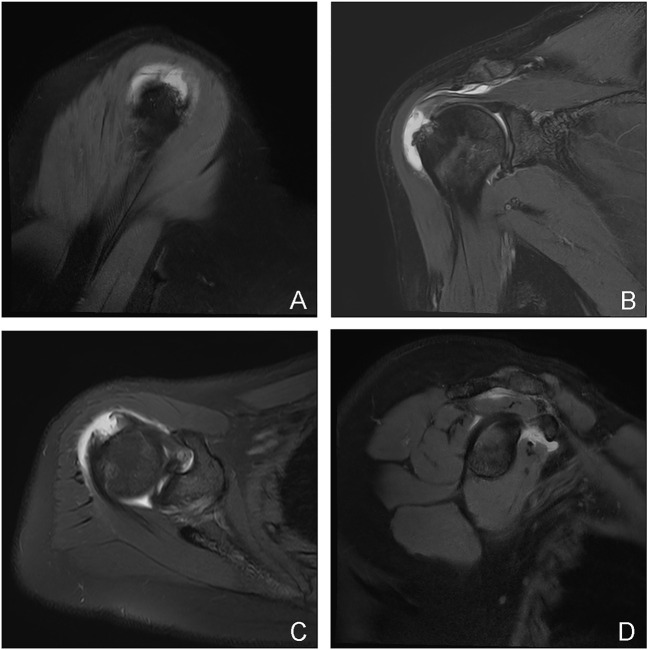
A typical case of a U-shaped tear: a 44-year-old woman with a localized full-thickness tear of the right shoulder, medial posterior to the supraspinatus tubercle insertion, with a “U"-shaped change **(A–C)**. **(A)** the oblique sagittal view of T2 lipid-suppressing sequence; **(B)** the axial view of proton density sequence; **(C)** the oblique coronal view of T2 lipid-suppressing sequence; **(D)** the oblique sagittal Y view of the rotator cuff from the T2 fat-suppressing sequence, showing mild atrophy of the supraspinatus and anterior infraspinatus with a little edema.

**FIGURE 3:**
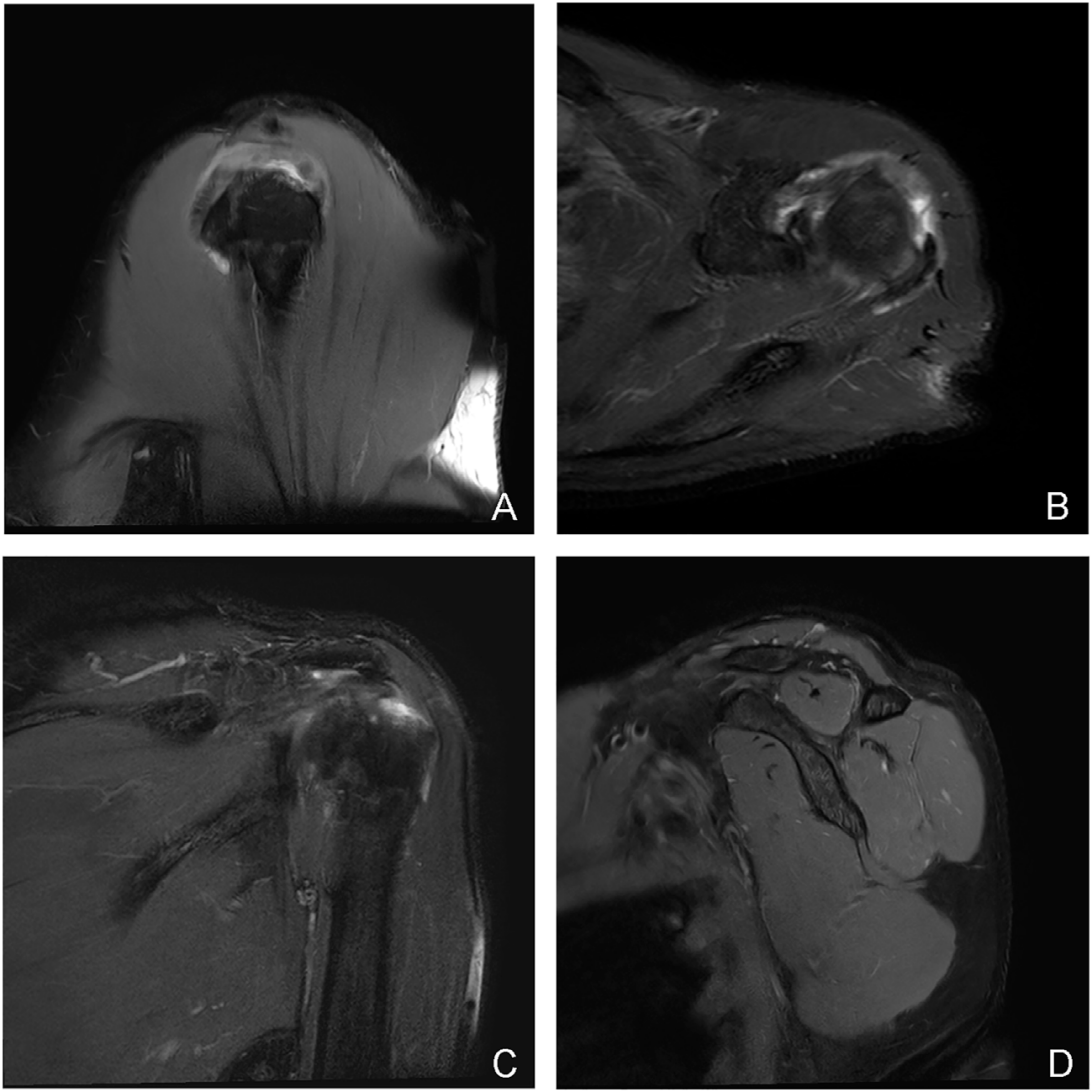
A typical case of crescent-shaped tear: a 51-year-old man with a “crescent” tear of the articular rim tendon in the left shoulder, at the insertion of the posterior and anterior tuberosities of the supraspinatus and infraspinatus **(A–C)**. **(A)** the oblique sagittal layer of T2 lipid-suppressed sequence; **(B)** the axial layer of proton density sequence; **(C)** the oblique coronal layer of T2 lipid-suppressed sequence; **(D)** the oblique sagittal layer of the rotator cuff Y-view of the T2 fat-suppressed sequence, showing the normal shape and signal of the four muscles that make up the rotator cuff.

**FIGURE 4:**
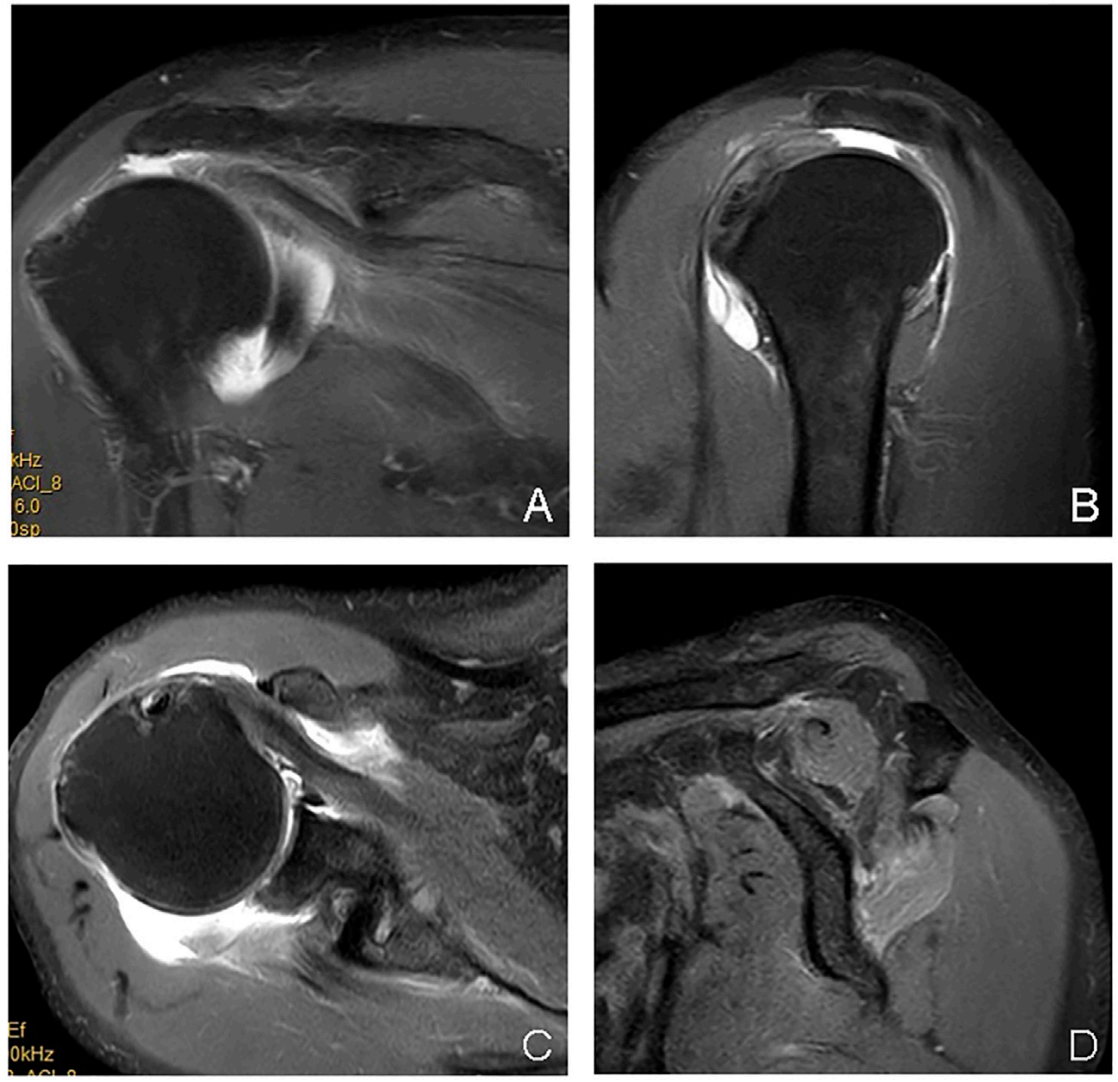
A typical case of the complete tear group: male, 70 years old, right shoulder joint, a complete tear of the supraspinatus and infraspinatus in the tubercle insertion area, the stump tendon had retracted **(A–C)**; supraspinatus and infraspinatus muscle atrophy with intramuscular fat deposition **(A–C)**. **(A)**: the oblique sagittal panel of T2 lipid-suppressing sequence; **(B)**: the axial panel of proton density sequence; **(C)** the oblique coronal panel of T2 lipid-suppressing sequence; **(D)** the rotator cuff Y view of the oblique sagittal panel of T2 fat-suppression sequence.

We divided the study population into three groups: 1) those involving L-shaped tears, i.e., a tear involving the anterior part of the supraspinatus tendon (*n* = 25) (Figure 1); 2) those with crescent/U-shaped tears, i.e., a tear that did not affect the anterior part of the supraspinatus tendon or the posterior part of the infraspinatus tendon (n = 10) (Figures 2, 3); 3) those with a complete tear of the entire posterior upper RC (n = 15) (Figure 4). Although the specific shapes of the crescent and the U-shaped tears are different, the locations of the crescent and the U-shaped tears are almost all in the middle region of the tuberosity insertion and the continuity of the corresponding edges of the insertion of the anterior and posterior tendon is normal. Crescent-shaped and U-shaped tears have high similarities in biomechanical analysis, pathological mechanism, and prognosis, so we grouped the two into one group for demonstration (Davidson and Burkhart, 2010).

This retrospective study was carried out in compliance with the ethical guidelines of international clinical research trials and the Helsinn Standard, and it has been approved by the Ethics Committee of our hospital to exempt the requirement of informed consent from patients.

### 2.2 Inclusion and exclusion criteria

The inclusion criteria were as follows: 1) Radiologically, the final diagnosis was a full-thickness supraspinatus tendon tear (STT); 2) the MRI report was reviewed twice by two senior musculoskeletal radiologists, and the diagnosis was confirmed as a full-thickness STT; 3) the full-thickness STT manifested as either L, U, or crescent-shaped, and the supraspinatus muscle and infraspinatus tendon were completely torn.

The exclusion criteria were as follows: 1) A previous RCR or shoulder replacement; 2) the loss of oblique sagittal T1 weighted sequence or poor image quality; 3) The full-thickness STT tear shape did not conform to the L, U, or crescent shapes, or a complete tear; 4) the patient had concurrent complications with abnormal lesions of the supraspinatus muscle.

### 2.3 Magnetic resonance imaging and image analysis

All shoulder MRI examinations were performed with 3.0T MRI, i.e., using a special shoulder coil. The scanning sequence included an oblique sagittal spin-echo sequence (SE), an oblique sagittal and oblique coronal gradient inversion recovery sequence/spin-echo gradient sequence, a T2 weighted fat suppression sequence, and an oblique coronal and axial proton density sequence. The slice thickness was 3 mm for all sequences.

**FIGURE 5:**
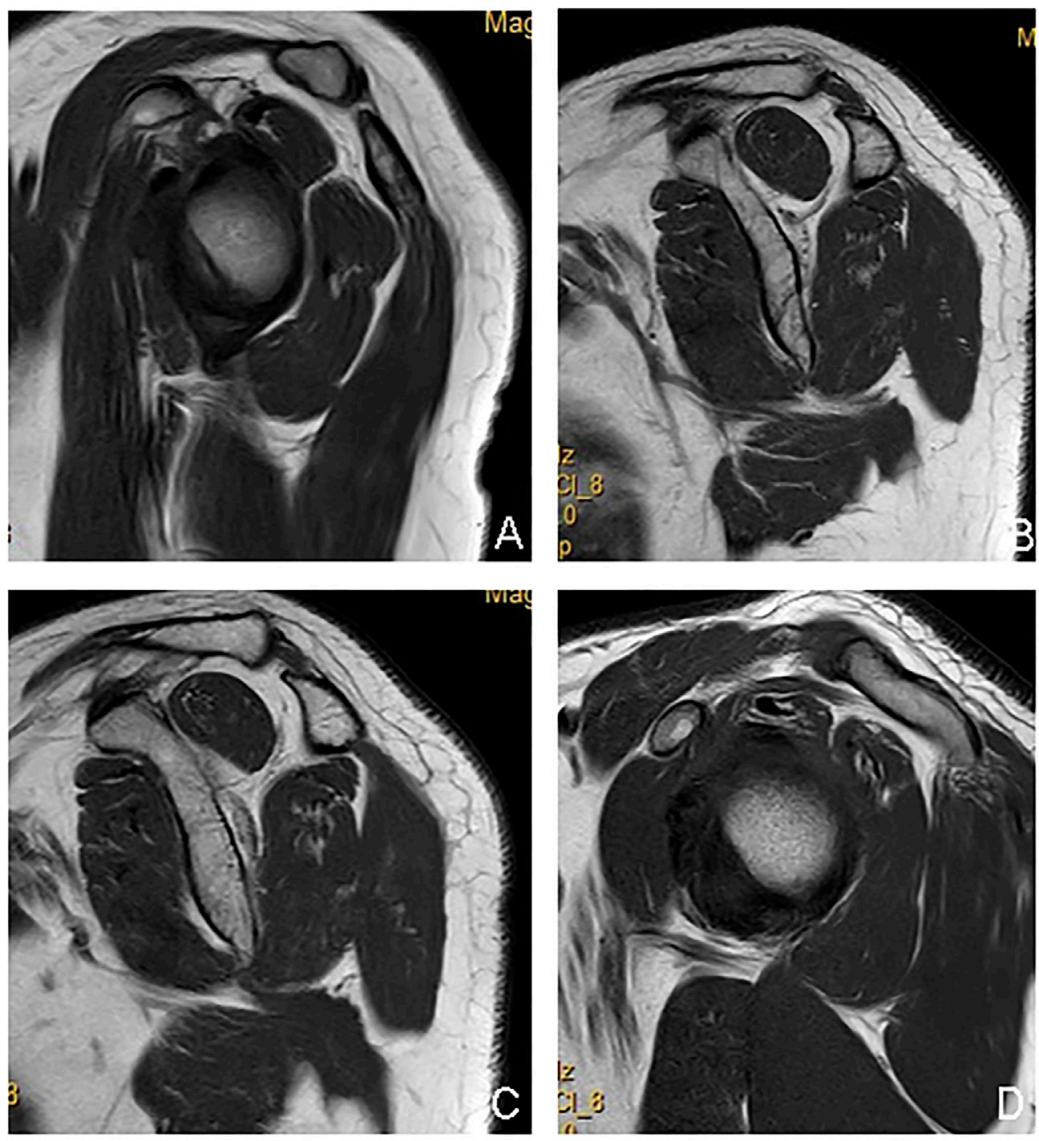
Goutallier grading display of rotator cuff Y-view (0–4 points), to evaluate the degree of abdominal atrophy and intramuscular fat deposition of the supraspinatus and infraspinatus muscles. **(A)** The shape of the supraspinatus and infraspinatus muscles is normal, and there is no fat signal inside, Goutallier = 0 points; **(B)** The shape of the supraspinatus and infraspinatus muscles is normal, and there is a fat streak signal in the supraspinatus muscle, Goutallier = 1 point; there are multiple fat signals in the infraspinatus muscle, but the muscle signal range is greater than the fat signal range, Goutallier = 2 points; **(C)** The supraspinatus muscle was slightly atrophied, and there were flaky fat signals inside, but the muscle signal range was greater than the fat signal range, Goutallier = 2 points; the infraspinatus muscle was slightly reduced in size, and multiple fat signals appeared inside the infraspinatus muscle, and the fat signal range accounted for about 1/2, Goutallier = 3 points; **(D)** Supraspinatus muscle atrophy, multiple fat signals appear in its interior, muscle signal range < fat signal range, Goutallier = 4 points; infraspinatus muscle is slightly reduced in size, multiple fat signals appear inside infraspinatus muscle, and fat signal range accounts for about 1/2, Goutallier = 3 points.

A blind analysis of the images was performed by two senior skeletal muscle-system imaging diagnostic physicians, where images were read from the PACS. The imaging sequences included oblique sagittal T1 and T2 weighted sequences corresponding to a Y-shaped view of each study object in the study (Figure 5). Then, the degree of intramuscular FI in the supraspinatus and infraspinatus muscles was independently evaluated according to the blind Goutallier grading method (Yoo et al., 2009; Jeong et al., 2017).

The Goutallier grades employed in this study were as follows: grade 0, no fat; grade 1, fat stripe; grade 2, muscle > fat; grade 3, muscle = fat; grade 4, muscle < fat (Mellado et al., 2005).

Two diagnostic radiologists retrospectively analyzed the MRI images of each shoulder included among the study population and measured the medial–lateral and anterior–posterior tear-range data of each full-thickness posterior upper RCT, before recording the tears according to the RCT shape defined by Davidson et al. (L, U, or crescent-shaped tears (Davidson and Burkhart, 2010; Sela et al., 2015). A complete tear was defined as a full-thickness tear involving the entire supraspinatus and infraspinatus tendons.

### 2.4 Observational outcome

The Goutallier grade was the primary outcome, and other clinical data were secondary outcomes such as age, male-to-female ratio, tear range, trauma history, symptom duration, etc.

All of the included clinical data were provided by the PACS of the clinical institution. These data included age, gender, trauma history, and the duration of corresponding clinical symptoms related to shoulder MRI, whether shoulder pain was present, and any shoulder instability and/or limited activity function; these factors were analyzed for the duration of symptoms either below or above 4 weeks.

### 2.5 Statistical analysis

Data were analyzed using the Stata 15.0 statistical software. The age, male-to-female ratio, tear range, trauma history, symptom duration (below or above 4 weeks), and the mean Goutallier grade of the supraspinatus muscle and the mean Goutallier grade of the infraspinatus muscle were compared using a chi-square test. The mean Goutallier grades of the supraspinatus and infraspinatus muscles were compared between groups using an unpaired *t*-test after the data were examined by Kolmogorov–Smirnov test. A Spearman’s rank correlation coefficient was used to evaluate the correlations among age, male percentage, tear range, trauma history, and the Goutallier grade of the supraspinatus and infraspinatus in each group. The reliability between the two assessors was determined by calculating the kappa value; *p* < 0.05 was considered statistically significant.

## 3 Results

**FIGURE 6:**
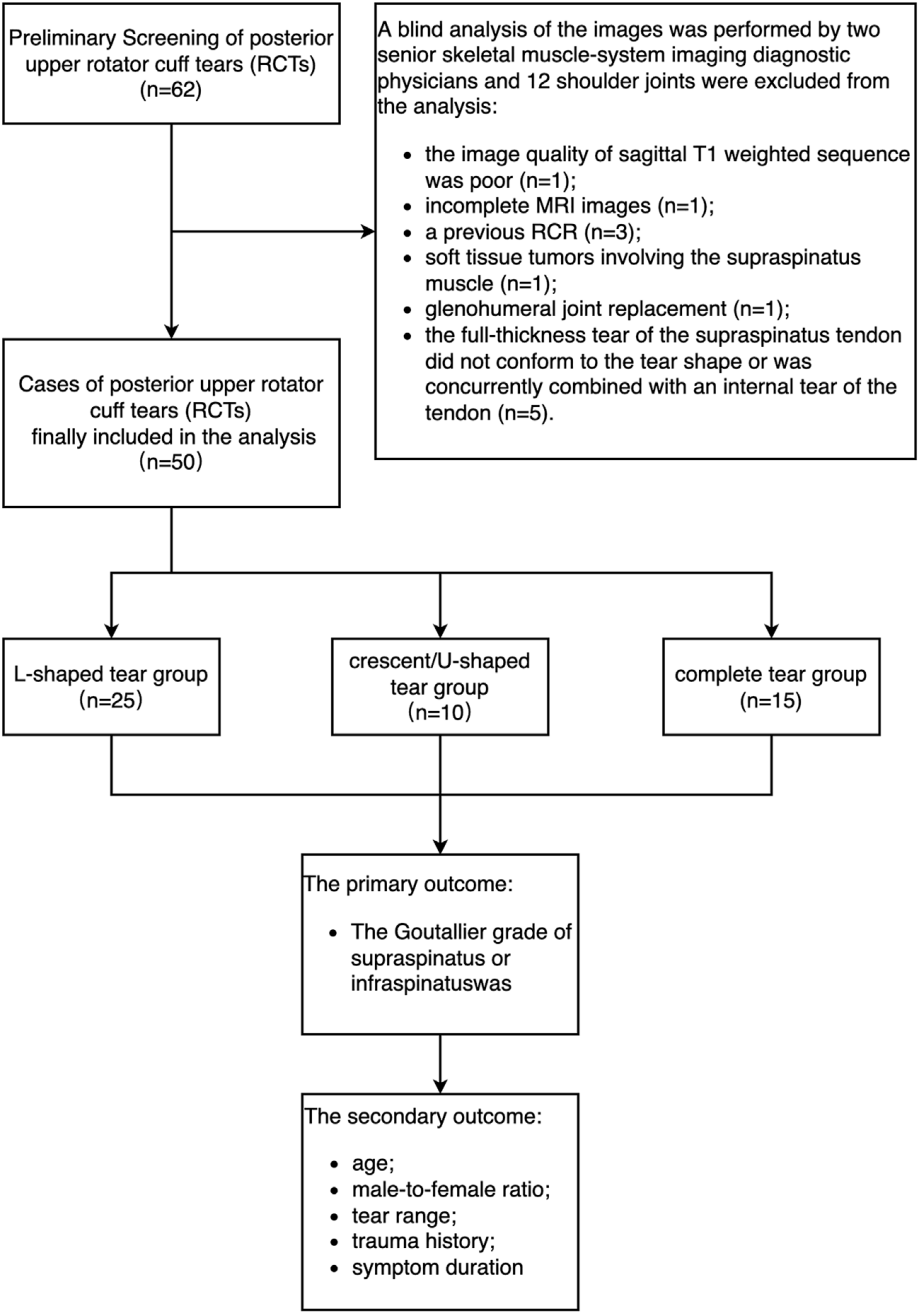
The flow-diagram of enrollment and observational outcomes.

From January 2019 to December 2021, the total of 62 shoulder joints diagnosed with full-thickness STT were all included in this study, among which 12 shoulder joints were excluded from the analysis. The reasons for this were as follows: the image quality of sagittal T1 weighted sequence was poor (*n* = 1); incomplete MRI images (*n* = 1); a previous RCR (*n* = 3); soft tissue tumors involving the supraspinatus muscle (*n* = 1); glenohumeral joint replacement (*n* = 1); the full-thickness tear of the supraspinatus tendon did not conform to the tear shape or was concurrently combined with an internal tear of the tendon (*n* = 5). Accordingly, a total of 50 shoulder joints were eligible in this study, including 66% male patients and 34% female patients. The age of the patients ranged between 27 and 78 years old, with an average age of 45.7 ± 6.9 years (Figure 6).

### 3.1 Comparisons of goutallier grade of supraspinatus and infraspinatus

There was a significant difference in the average Goutallier grade of the infraspinatus among groups (*p* = 0.036) but no significant difference was observed in the average Goutallier grade of the supraspinatus among the groups (*p* = 0.356), where the average Goutallier grade of the supraspinatus and infraspinatus in the complete tear group (*p* = 0.053) closely matched the statistical difference level; there was no significant difference between the L-shaped (*p* = 0.126) and crescent/L-shaped tear groups concerning the average Goutallier grade of the supraspinatus and infraspinatus (*p* = 0.313). The average Goutallier score of the infraspinatus was higher in the three groups; the average Goutallier score of the supraspinatus and infraspinatus in the L-shaped tear group was the lowest, and the average Goutallier score of the supraspinatus and infraspinatus in the complete tear group was the highest. (Table 1).

**TABLE 1:** Comparisons of Goutallier grade of supraspinatus and infraspinatus.

Goutallier grade	L-shaped group (*n* = 25)	Crescent/U-shaped group (*n* = 10)	Complete group (*n* = 15)	*p*-value (between groups)
supraspinatus	0.79 ± 0.88	1.01 ± 0.97	1.35 ± 0.78	0.356
infraspinatus	1.26 ± 0.89	1.47 ± 1.32	2.15 ± 0.98	0.036
*p*-value (Within groups)	0.126	0.313	0.053	

### 3.2 An analysis and summary of the clinical data of all the subjects in each group

There was a significant difference in the tear range among the groups (*p* < 0.001). The ranges of all full-thickness RCTs were <5 cm in the crescent/U-tear group and <3 cm in the L-shaped tear group; most of the RCTs with a range of <1 cm belonged to the L-shaped tear group, while all the RCTs with a range of ≥5 cm belonged to the complete tear group.

There was a significant difference in the participants’ trauma histories among the groups (*p* = 0.003). The number of patients with trauma histories in the complete tear group was the largest, and there was no significant difference in age, gender, or symptom duration among the groups. (Table 2).

**TABLE 2:** An analysis and summary of the clinical data of all the subjects in each group.

Items	L-shaped group (*n* = 25)	Crescent/U-shaped group (*n* = 10)<	Complete group (*n* = 15)	*p*-value
Age (years)	52.56 ± 8.16	53.69 ± 9.36	51.15 ± 8.96	0.689
Male %	46.8%	45.3%	69.6%	0.076
Tear range, n (%)				<0.001
<1 cm	9 (36.0%)	2 (20.0%)	0 (0.0%)	
1–3 cm	16 (64.0%)	3 (30.0%)	0 (0.0%)	
3–5 cm	0 (0.0%)	5 (50.0%)	9 (60.0%)	
≥5 cm	0 (0.0%)	0 (0.0%)	6 (40.0%)	
Trauma history, n (%)				0.003
Yes	8 (32.0%)	5 (50.0%)	12 (80.0%)	
No	17 (68.0%)	5 (50.0%)	3 (20.0%)	
Duration ≤ 4 weeks, n (%)				0.156
Yes	4 (16.0%)	3 (30.0%)	4 (26.7%)	
No	21 (84.0%)	7 (70.0%)	11 (73.3%)	

### 3.3 Correlation analysis between goutallier grade and other clinical data in three groups

In the L-shaped tear group, there was a medium-to-high correlation between the percentage of male participants and the average Goutallier grade of the infraspinatus (*p* = 0.004, Table 3). There was a near-mild correlation between the average Goutallier grade of the infraspinatus with age (*p* = 0.061) and tear range (*p* = 0.059) and between the average Goutallier grade of the supraspinatus and the percentage of male participants (*p* = 0.071).

**TABLE 3:** Correlation analysis of the average Goutallier grade of supraspinatus and infraspinatus in the L-shaped tear group.

	Supraspinatus		Infraspinatus	
	rs	P	rs	P
Age (years)	0.254	0.152	0.351	0.061
Male %	0.336	0.071	0.541	0.004
Tear range	0.269	0.279	0.387	0.059
Trauma history	0.296	0.139	0.156	0.871

In the crescent/U-shaped tear group, there was a significant correlation between age and the average Goutallier grade of the supraspinatus (*p* = 0.029) and the average Goutallier grade of the infraspinatus (*p* = 0.032) (Table 4).

**TABLE 4:** Correlation analysis of the average Goutallier grade of supraspinatus muscle and infraspinatus muscle in the crescent/U-shaped tear group.

	Supraspinatus		Infraspinatus	
	rs	P	rs	P
Age (years)	0.720	0.029	0.713	0.032
Male %	0.489	0.181	0.556	0.139
Tear range	0.521	0.149	0.526	0.148
Trauma history	0.431	0.249	0.521	0.152

In the complete tear group, there was a significant correlation between the tear range and the average Goutallier grade of the supraspinatus (*p* = 0.001) and the average Goutallier grade of the infraspinatus (*p* = 0.001) (Table 5). The inter-observer reliability of the Goutallier grades of the supraspinatus (kappa, 0.489) and the infraspinatus (kappa, 0.424) was moderate.

**TABLE 5:** Correlation analysis of the average Goutallier grade of supraspinatus and infraspinatus in the complete tear group.

	Supraspinatus		Infraspinatus	
	rs	P	rs	P
Age (years)	0.258	0.385	0.231	0.448
Male %	−0.101	0.745	−0.220	0.456
Tear range	0.801	0.001	0.802	0.001
Trauma history	0.103	0.740	0.223	0.461

## 4 Discussion

The present study revealed that the shape and size of a full-thickness posterior upper RCT positively correlated with FI. The L-shaped group, crescent/U-shaped group, and the complete tear group showed different average Goutallier grades in the posterior upper RCT. In the crescent/U-shaped tear group, age was highly correlated with the average Goutallier grades of the supraspinatus and the infraspinatus. In the complete tear group, the tear range was highly correlated with the average Goutallier grades of the supraspinatus and the infraspinatus. In the present study, there was a significant difference in the tear range among the groups, where the complete tear group showed the largest damage range to the posterior upper RCT, and for which the corresponding average Goutallier grade score was the highest. Although there was a significant difference in only the Goutallier grade of the infraspinatus, we still assumed that the RCT range was highly and positively correlated with the average Goutallier grade of the supraspinatus and the infraspinatus.

Existing studies revealed that, compared with RCTs in other regions, tears in the anterior supraspinatus tendon were related to a higher FI of the supraspinatus (Updegrove et al., 2015; Collin et al., 2019). Researchers speculated that the tendon histology and function of the anterior supraspinatus tendon are different from the rest of the RC (Osti et al., 2017). Kim et al. revealed that all full-thickness RCTs, including small-scale tears, would cause more severe supraspinatus FI than expected when the anterior supraspinatus tendon was involved, and relatively less FI when the anterior supraspinatus tendon was not involved (Kim et al., 2010).

Theoretically, the crescent/U-shaped RCT, rather than the anterior tear involving the supraspinatus tendon, will reduce a harmful biomechanical load throughout the complete anterior and posterior RC. Namdari et al. compared the shoulder joints of small and medium-sized STTs with and without anterior supraspinatus tendon participation; the RCT involving the anterior showed high FI of the supraspinatus via ultrasonography (Namdari et al., 2014). There was no significant difference in the clinical parameters (such as baseline pain, functional recovery, or prognosis) after arthroscopic RCR. In the ultrasound examination of the anterior STT group, the FI of the infraspinatus tended to increase but the difference was not statistically significant (Namdari et al., 2014; Chen et al., 2020).

In the present study, the average Goutallier grade of the supraspinatus and the infraspinatus in the L-shaped tear group was lower than in the crescent/U-shaped tear group, which may have been due to the smaller tear range in the L-shaped tear group.

The RC forms an important structural complex with the leading edge of the supraspinatus tendon, where its anatomical structure helps to transfer force from the supraspinatus tendon to the proximal humerus (Kim et al., 2010; Updegrove et al., 2015). It is carried by the entire RC to provide stress-shielding protection to the crescent region of the posterior upper RC (Podgórski et al., 2019). Compared with an anterior tear of the supraspinatus tendon, a full-thickness tear involving the RC-anterior supraspinatus tendon complex is assumed to be closely related to RC dysfunction (Kim et al., 2010; Updegrove et al., 2015). Mesiha et al. revealed that the integrity of the RC cuff-anterior supraspinatus tendon complex is the main structure that ensures the normal distribution of biomechanical load from the scapula to the proximal humerus (Mesiha et al., 2013). These study results revealed that compared with a crescent RCT with the same tear range, a full-thickness tear involving the anterior supraspinatus tendon was associated with larger tendon tears and displacement and mechanical changes in the stress area; it is speculated that a tear in the RC-anterior supraspinatus tendon complex may be related to serious clinical symptoms and joint function damage within a short time, e.g., severe pain and RC muscle dysfunction, and a tendency for the progression and aggravation of an RCT at a later stage (Mesiha et al., 2013; Collin et al., 2020).

Disagreement remains as to where the posterior upper RCT begins. Some theories suggest that it begins at the center of the crescent area and in the anterior supraspinatus tendon (Kim et al., 2010; Namdari et al., 2014). The researchers speculated that age played an important role in the mechanical load transfer from the scapula to the humerus through the RC-anterior supraspinatus tendon complex. Podgórski et al. illustrated two different age-related RCT patterns. 1) Young patients without RC stress-shielding due to the presence of a thick and strong RC crescent part (the crescent dominant mode). 2) Elderly patients with a weak RC crescent part due to age-related tendon degeneration, which requires mechanical loading of the RC (the RC dominant mode) (Podgórski et al., 2019). One hypothesis posited that RC hypertrophy was an adaptation to age-related RC crescent thinning (as opposed to a traumatic crescent tear) and that the late degeneration of the central RC of its insertion part was significantly correlated with age (Podgórski et al., 2019). The present study revealed that in the crescent/U-shaped tear group, age was highly correlated with the average Goutallier grade of the supraspinatus and the infraspinatus, which supports the above hypothesis. The theory proposes that because the key bearing function of the RC-anterior supraspinatus tendon complex is lost, an RC-leading shoulder is more important for elderly patients (Podgórski et al., 2019).

Infraspinatus FI is not proportional to the range of full-thickness tears of the infraspinatus tendon observed via MRI. Mochizuki et al. revealed in an autopsy study that the footprint of the insertion part of the infraspinatus tendon was larger than previously assumed and that its insertion occurred further forward into the articular surface of the upper part of the greater tubercle of the humerus; therefore, the insertion range of the greater nodules of the humerus of the supraspinatus tendon was observed to be much smaller than previously thought (Mochizuki et al., 2008). In the present study, compared with the supraspinatus, the average Goutallier score of the infraspinatus was higher, although there was a significant positive correlation trend only in the complete-tear group.

Because shoulder RCT patterns affect the development of surgical planning, in current clinical practice, most RCR surgeries are performed arthroscopically. Crescent tears are typically repaired with direct tendons at the greater nodules of the humerus, while U-shaped and smaller L-shaped tears require tendon edge convergence and side-to-side tendon repair before the broken tendon can be directly anchored to the bone insertion. When the supraspinatus and the infraspinatus tendons have a wider range of L-shaped tears and/or complete tears, it may not be possible to restore all the natural footprints of the posterior upper RC through surgery. These large-range RCTs may require more extensive soft tissue release or tension-free repair (Mihata et al., 2015; Collin et al., 2020). In addition, when tendon fibers are severely denatured, an RCT repair may require allografts or joint capsule reconstruction. In general, chronic large-area RCTs with FI have a poorer prognosis for shoulder function impairment than acute small RCTs without FI (Mihata et al., 2015; Podgórski et al., 2019).

This study includes some limitations. Because of its retrospective nature, only relevant clinical information in the PACS of our institution could be retrieved. The dominant hand and the range and intensity concerning daily activities involving the shoulder of the study subjects were unclear. Our conclusions may not be applicable to other RC tear types that were not included in this study. There is a lack of surgical confirmation regarding. The study sample was also relatively small. In the future, large-scale studies should be conducted to verify the results of the current research and evaluate whether statistical differences exist among other types of full-thickness RCTs. Future big data research projects are necessary to determine whether there is a clinical correlation between different RCT patterns, FI, and postoperative results.

## 5 Conclusion

The type of posterior upper RCT correlates with the degree of FI. There is a positive correlation between the FI of crescent/U-shaped full-thickness RCTs and age. Additionally, the range of complete tears in the posterior upper RC has a positive correlation with FI.

## Data Availability

The original contributions presented in the study are included in the article/supplementary material, further inquiries can be directed to the corresponding author.
